# MicroRNA-1258 suppresses oxidative stress and inflammation in septic acute lung injury through the Pknox1-regulated TGF-β1/SMAD3 cascade

**DOI:** 10.1016/j.clinsp.2024.100354

**Published:** 2024-04-18

**Authors:** XiaoMeng Xu, XiaoHong Xu, JinLiang Cao, LuoYang Ruan

**Affiliations:** a Guangzhou Hospital of Integrated Traditional and West Medicine, Department of Anesthesiology, Guangzhou City, Guangdong Province, China; b Guangzhou Hospital of Integrated Traditional and West Medicine, Department of Pediatrics, Guangzhou City, Guangdong Province, China

**Keywords:** MicroRNA-1258, Pknox1, Sepsis, Acute lung injury, Transforming growth factor β1/SMAD3 pathway, Inflammation, Oxidative stress

## Abstract

**Aim:**

The study was to clarify the mechanism of miR-1258 targeting Prep1 (pKnox1) to control Transforming Growth Factor β1 (TGF-β1)/SMAD3 pathway in septic Acute Lung Injury (ALI)-induced oxidative stress and inflammation.

**Methods:**

BEAS-2B cells and C57BL/6 mice were used to make *in vitro* and *in vivo* septic ALI models, respectively. miR-1258 expression was checked by RT-qPCR. After transfection in the *in vitro* experimental model, inflammation, oxidative stress, viability, and apoptosis were observed through ELISA, MTT, and flow cytometry.

**Results:**

In the *in vivo* model after miR-1258 overexpression treatment, inflammation, oxidative stress, and lung injury were further investigated. The targeting relationship between miR-1258 and Pknox1 was tested. Low miR-1258 was expressed in septic ALI patients, LPS-treated BEAS-2B cells, and mice. Upregulated miR-1258 prevented inflammation, oxidative stress, and apoptosis but enhanced the viability of LPS-treated BEAS-2B cells. The impact of upregulated miR-1258 on LPS-treated BEAS-2B cells was mitigated by inhibiting Pknox1 expression. MiR-1258 overexpression had the alleviating effects on inflammation, oxidative stress, and lung injury of LPS-injured mice through suppressing Pknox1 expression and TGF-β1/SMAD3 cascade activation.

**Conclusions:**

The study concludes that miR-1258 suppresses oxidative stress and inflammation in septic ALI through the Pknox1-regulated TGF-β1/SMAD3 cascade.

## Highlights

• MiR-1258 is downregulated in septic ALI.
• MiR-1258 inhibits inflammation and oxidative stress via Pknox1 in ALI.
• MiR-1258 targets Pknox1 to control TGF-β1/SMAD3 cascade.

## Introduction

Sepsis is a systemic inflammatory response syndrome caused by severe infection.^1^ Sepsis often leads to multiple organ failures and even death.^2^ The lungs are the most common failing organ in sepsis and is also the primary site with the highest frequency of infection.^3^ Under septic conditions, excessive inflammation and apoptosis lead to the destruction of alveolar epithelial cells, increased epithelial permeability, and the influx of edema fluid into the alveolar space, ultimately leading to Acute Lung Injury (ALI).^4^ However, there are no specific drugs or treatments for sepsis and septic ALI.

The inflammatory response has been indicated in the pathogenesis of septic ALI, and this process is associated with the upregulation of inflammatory cytokines and chemokines (Transforming gGrowth Factor-β1 [TGF-β1], Interleukin [IL]-1β, IL-6, IL-13, and Tumor Necrosis Factor [TNF]-α).^5^^,^^6^ TGF-β1 is an important inflammatory cytokine involved in various pathophysiological processes. Although the underlying mechanism of TGF-β-mediated ALI remains unclear, studies suggest that TGF-β1 may enhance ALI by increasing pulmonary microvascular endothelial and alveolar epithelial cell permeability and promoting actin stress fiber formation.^7^ Elevated TGF-β1 in ALI mice is involved in the late stages and leads to lung injury in the early stages of disease progression.^8^ In addition, oxidative stress is also one of the key pathological processes of sepsis-related ALI.^9^ TGF-β1 is associated with oxidative stress in various diseases,^10^ indicating the importance of TGF-β1 in septic ALI.

MicroRNAs (miRNAs) participate in various cellular processes, including inflammation, oxidative stress, and apoptosis.^11^^,^^12^ Pathologically, miRNAs are associated with human diseases, including ALI.^13^ It has been previously documented that miRNAs such as miR-490 and miR-494 ameliorate septic ALI.^14^^,^^15^ MiR-1258 has a tumor suppressor role by targeting heparanase in gastric, breast and non-small cell lung cancers,^16^ and is implicated in cancer immune evasion and systemic inflammation.^17^^,^^18^ Nevertheless, the role of miR-1258 in ALI remains unreported.

Lipopolysaccharide (LPS) is an endotoxin that activates the inflammatory signaling pathway through Toll-Like Receptor 4 (TLR4), induces a large number of inflammatory cytokines, and recruits a wide range of macrophages and neutrophils in the lung. LPS-induced in vivo and in vitro models have been recognized as classic models of acute lung injury due to sepsis.^19^ The present study hypothesized that miR-1258 alleviates oxidative stress and inflammation in septic ALI through the Pknox1-regulated TGF-β1/SMAD3 cascade and aimed to investigate the role and mechanisms of miR-1258 in septic ALI. The objectives included investigating miR-1258 expression in patients and septic ALI models and understanding the function and mechanism of miR-1258 in LPS-induced inflammation and oxidative stress in vitro. The study also aimed to determine whether MiR-1258 targets Pknox1-regulated TGF-β1/SMAD3 cascade and if miR-1258 prevents LPS-induced inflammation and oxidative stress *in vivo.*

## Materials and methods

### Serum specimen

Forty-four patients with septic ALI admitted to Guangzhou Hospital of Integrated Traditional and West Medicine from July 2019 to July 2020 were selected, including 27 males and 17 females aged 33 to 70 years old, with an average of (53.35 ± 10.01) years old. Another 44 healthy donors with no smoking history were selected, including 24 males and 20 females; the ages ranged from 27 to 70 years old, with an average of (50.13 ± 9.14) years old. Gender and age had no significant difference (p > 0.05). Sepsis and ALI were diagnosed according to the corresponding international criteria. The ALI diagnosis criteria used included an acute onset, PaO_2_/FiO_2_ ≤ 200 mm Hg, and bilateral infiltrates seen on the frontal chest radiograph. The clinical symptoms used for diagnosis included dyspnea, tachypnea, production of sputum, hypoxia, and pleuritic chest pain.^20^ Blood samples were obtained and centrifuged at 8000 × g for 3 min.

### Cell culture and transfection

Human normal lung epithelial cell line BEAS-2B (ATCC) maintained in DMEM containing 10 % fetal bovine serum were transfected with mimic-CTR, miR-1258 mimic, si-CTR, si-Pknox1 and oe-Pknox1 (Genepharma, Shanghai, China) using Lipofectamine 3000 (Invitrogen, CA, USA). The medium was purchased from Gibco (CA, USA). A Lipopolysaccharide (LPS)-induced model was established after 24h, cell exposure to 1.0 μg/mL LPS for an additional 24h (Sigma-Aldrich, MO, USA).

### Experimental animal

Adult healthy male C57BL/6 mice (25s∼30g, 6∼8 weeks-old; Beijing Vital River Laboratory Animal Technology Co., Ltd., Beijing, China) were fed with standard feed and purified water under the conditions of 61 % air humidity, 21∼26°C temperature, and 12h light/darkness.

LPS intravenous injection to induce septicemic lung injury is the most widely used method for modeling ALI.^21^^,^^22^ After intraperitoneal injection of LPS at 10 mg/kg, increased respiratory rate, chills, standing hair, decreased activity, and watery stool indicated the successful induction of ALI. Normal mice were injected with 0.20 mL of normal saline.

### Animal treatment

The mice were divided into 4 groups, with 10 mice in each group. Except for the Sham and ALI groups, mice in the agomir-CTR group and miR-1258 agomir group were injected intravenously with agomir-CTR and miR-1258 agomir (GenePharma) for 3 consecutive days prior to LPS induction. After euthanizing the mice, lung tissue and blood from the inner canthus were collected for testing.

All animal experiments complied with the ARRIVE guidelines and performed in accordance with the National Institutes of Health Guide for the Care and Use of Laboratory Animals. The experiments were approved by the Institutional Animal Care and Use Committee of Guangzhou Hospital of Integrated Traditional and West Medicine.

### Inflammatory factor detection

Mouse serum was collected by centrifugation, and the BEAS-2B cell culture supernatant was amassed. TNF-α, IL-6, and IL-1β in serum and cell supernatant were detected using enzyme-linked immunosorbent assay kits (ELISA, R&D company). The absorbance was determined at an optical density of 490 nm on a microplate reader.

### Oxidant indicator detection

BEAS-2B cells were suspended in 0.3 mL PBS and disrupted on ice by ultrasound to extract total intracellular protein. Lung tissues were prepared into tissue homogenate using normal saline. SOD activity^23^ and MDA and GSH contents were measured by kits (Nanjing JianCheng Bioengineering Institute, Nanjing, China).

### MTT assay

The single-cell suspension of BEAS-2B was cultured in the 96-well plate at 2 × 10^8^/L, 100 μL/well. The medium was replaced with fresh DMEM after 48h and incubated for an additional 12h. Next, MTT (5 g/L, 10 μL/well) was added, forming Formanzan, which was then dissolved by Formanzan lysis solution (100 μL/well). Finally, optical density_490 nm_ was measured.

### AnnexinV-FITC/PI double staining

BEAS-2B cells were plated in the 6-well plate at 2 × 10^8^/L for 24h. Later, adhered cells were detached and collected by centrifugation at 1500 r/min for 10 min. Next, after adding 400 μL Annexin V binding solution, BEAS-2B cells were incubated with 5 μL AnnexinV-FITC solution and 10 μL PI solution, followed by a flow cytometry test and WinMDI software analysis.

### Hematoxylin-eosin (H&E) staining

Mouse lung tissues were made into 5 μm slices and embedded into paraffin. Paraffinized sections were dehydrated with conventional gradient alcohol, cleared with xylene, stained with hematoxylin, differentiated with 1 % hydrochloric acid alcohol, and immersed in 1 % ammonia. After counter-staining with 1 % eosin solution, the slices were treated with conventional dehydration and permeability, sealed, and viewed under a microscope (Olympus, Tokyo, Japan).^23^

### Quantitative Real Time PCR detection

Lung tissues of mice in all groups, LPS-induced BEAS-2B cells, and control cells were taken after the above treatment and lysed, and total RNA was extracted by one-step method with Trizol (Invitrogen, CA, USA), of which the concentration and quality were determined by NanoDrop2000 (Thermo Fisher Scientific, USA). According to the Rever Tra Aceq PCR RT Kit (TOYOBO), 500 ng of RNA was loaded in the reverse transcription reaction system, and the products were processed according to the protocol of the Sybrgreen kit (Takara). MiR-1258 expression was examined using a Bulge-LoopTM miRNA kit (RuiBo, China) with U6 as a control. Primers listed in Table 1 were synthesized by BGI (Shenzhen, China), among which U6 and GAPDH were considered internal controls. Data were analyzed using the 2^−ΔΔCt^ method.^24^ All experiments were repeated three times.

**Table 1:** Sequences in PCR.

Gene	Sequences
miR-1258	RT: 5’-GTCGTATCCAGTGCAGGGTCCGAGGTATTCGCACTGGATACGACTTCCAC-3’
	R: 5’-ATCCAGTGCAGGGTCCGAGG-3’
	F: 5’- GCGGCGGAGTTAGGATTAGGTC-3’
Pknox1	F: 5’-AGCAGGCCATTTATAGGCATC-3’
	R: 5’-TCACCATTAGGTTGTCAGTTTCC-3’
U6	F: 5’-ATTGGAACGATACAGAGAAGATT-3’
	R: 5’-GGAACGCTTCACGAATTTG-3’
GAPDH	F: 5’-ACGGCAAGTTCAACGGCACAG-3’
	R: 5’-GACGCCAGTAGACTCCACGACA-3’

Note: miR-1258, MicroRNA-1258; Pknox1, Prep1; GAPDH, Glyceraldehyde-3-phosphate dehydrogenase.

### Western blot

Tissues and cells were processed to obtain total protein, and the protein concentration was determined by a BCA kit (Boster, Hubei, China). Proteins were separated by 10 % polyacrylamide gel electrophoresis, followed by immunoblotting onto PVDF membrane and blocking with 5 % BSA. Primary antibodies; TGF-β1 (Santa Cruz Biotechnology, 1:1000), Smad3, p-Smad3 (CST, 1:1000), and GAPDH (Abcam, 1:3000). The membrane was incubated with the corresponding secondary antibodies (MT-Bio, Shanghai, China). Finally, a chemiluminescence reagent was added to the membrane, and band development was observed using GELDOCEZIMAGER (Bio-rad, CA, USA). Band analysis was done using ImageJ software.

### Luciferase reporter gene assay

The binding of miR-1258 and Pknox1 was predicted using the bioinformatics software https://cm.jefferson.edu/rna22. MiR-1258 was found to bind to Pknox1 at the 3′UTR. Based on that, Pknox1 3′UTR wild-type plasmid (Pknox1 3′UTR-WT, with miR-1258 binding site) was synthesized, and Pknox1 3′UTR-MUT (with a mutated binding site). Lipofectamine 2000 (Invitrogen) was used to transfect BEAS-2B cells when 70 % confluence was achieved. The Pknox1 3′UTR-WT or Pknox1 3′UTR-MUT was co-transfected with miR-1258 to mimic or mimic NC. The luciferase activity was measured with the Dual-Luciferase Reporter Assay System kit (Promega, WI, USA).

### Statistical analysis

GraphPad Prism 8 was applied to statistical analysis. Data were presented as mean ± standard deviation. Normally distributed data were assessed by *t*-test, and otherwise by One-Way analysis of variance and Tukey's method; p was a two-sided test, and *p* < 0.05 was considered statistically significant.

## Results

### MiR-1258 is downregulated in patients and in in vitro septic ALI models

**Fig. 1:**
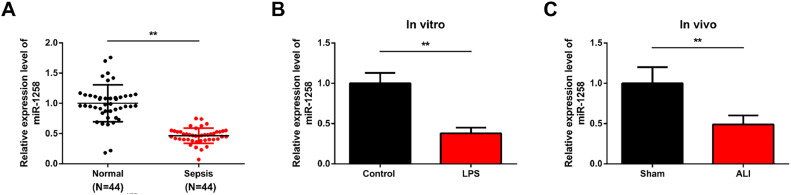
miR-1258 downregulation in clinical and experimental septic ALI. MiR-1258 expression in clinical serum samples (A, *n* = 44), LPS-treated BEAS-2B cells (B, Repetitio *n =* 3) and lung tissue of LPS-treated mice (C, *n =* 10). * *p* 0.05, ** *p <* 0.01. Data were presented as mean ± standard deviation.

There are currently no specific drugs or treatments for sepsis and septic ALI. The present study aimed to investigate miR-1258 in oxidative stress and inflammation in septic ALI. The objectives included investigating miR-1258 levels in septic ALI and understanding its function and mechanism in LPS-induced inflammation and oxidative stress. The study also aimed to determine whether miR-1258 targets Pknox1-regukated TGF-β1/SMAD3 cascade and if miR-1258 prevents LPS-induced inflammation and oxidative stress in vivo. miR-1258 expression was significantly reduced in ALI patients (Fig. 1A). RT-qPCR results confirmed a significant miR-1258 suppression in LPS-treated cells (Fig. 1B) and LPS-induced animals (Fig. 1C). These observations confirmed that miR-1258 is downregulated in patients, LPS-induced cells, and septic ALI models.

### MiR-1258 inhibits LPS-induced inflammation and oxidative stress via Pknox1 in vitro

**Fig. 2:**
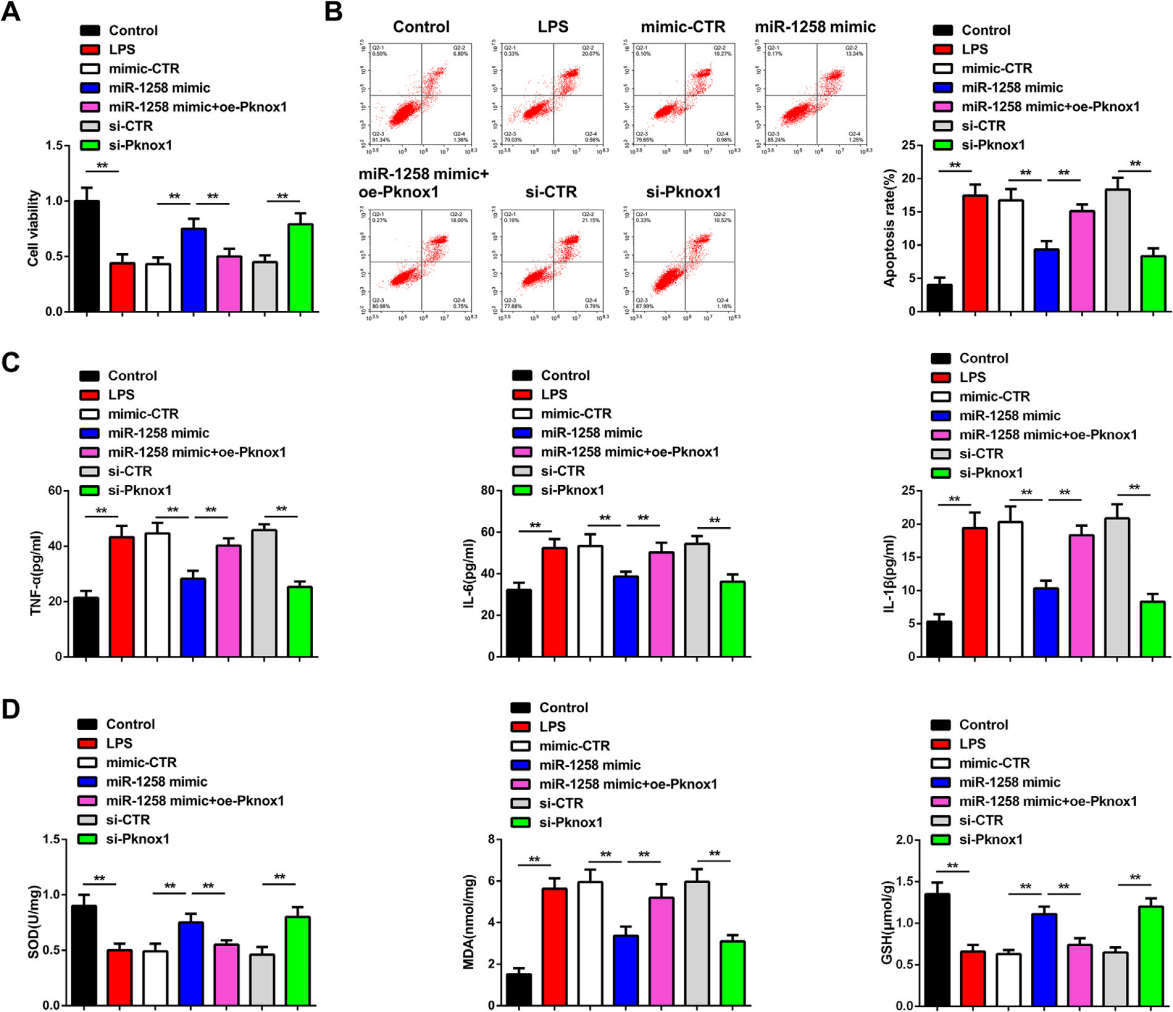
miR-1258 prevents LPS-induced inflammation and oxidative stress via Pknox1 *in vitro*. Cell viability (A), apoptosis rate (B), inflammatory factor concentrations (C), contents of oxidative stress-related indicators (D); Repetitio *n =* 3; Data were presented as mean ± standard deviation.

When analyzing the mechanism of miR-1258 in septic ALI, the BEAS-2B cells were transfected with LPS, miR-1258 mimic, mimic-CTR, miR-1258 mimic+oe-Pknox1, si-CTR or si-Knox1. The MTT assays confirmed significantly reduced cell viabilities in the LPS, mimic-CTR, miR-1258 mimic+oe-Pknox1, and si-CTR, but remarkably high cell viabilities in miR-1258 mimic and si-Pknox1 cells compared to the control group, as shown in Fig. 2A. Flow cytometry experiments confirmed increased apoptosis in LPS, mimic-CTR, miR-1258 mimic+oe-Pknox1, and si-Pknox1 compared to the miR-1258 mimic and si-Pknox1 (Fig. 2B). ELISA experiments confirmed significantly increased TNF-α, IL-6, and IL-1β in LPS, mimic-CTR, miR-1258 mimic+oe-Pknox1, and si-Pknox1 compared to the miR-1258 mimic and si-Pknox1, as shown in Fig. 2C. Oxidant indicators detection experiments confirmed significantly reduced SOD and GSH expressions in LPS, mimic-CTR, miR-1258 mimic+oe-Pknox1, and si-Pknox1 compared to the miR-1258 mimic and si-Pknox1. However, MDA was significantly increased in LPS, mimic-CTR, miR-1258 mimic+oe-Pknox1, and si-Pknox1 compared to the miR-1258 mimic and si-Pknox1 (Fig. 2D). These observations demonstrate that MiR-1258 alleviates LPS-induced inflammation and oxidative stress via Pknox1 *in vitro.*

### MiR-1258 targets Pknox1 to regulate TGF-β1/SMAD3 cascade

**Fig. 3:**
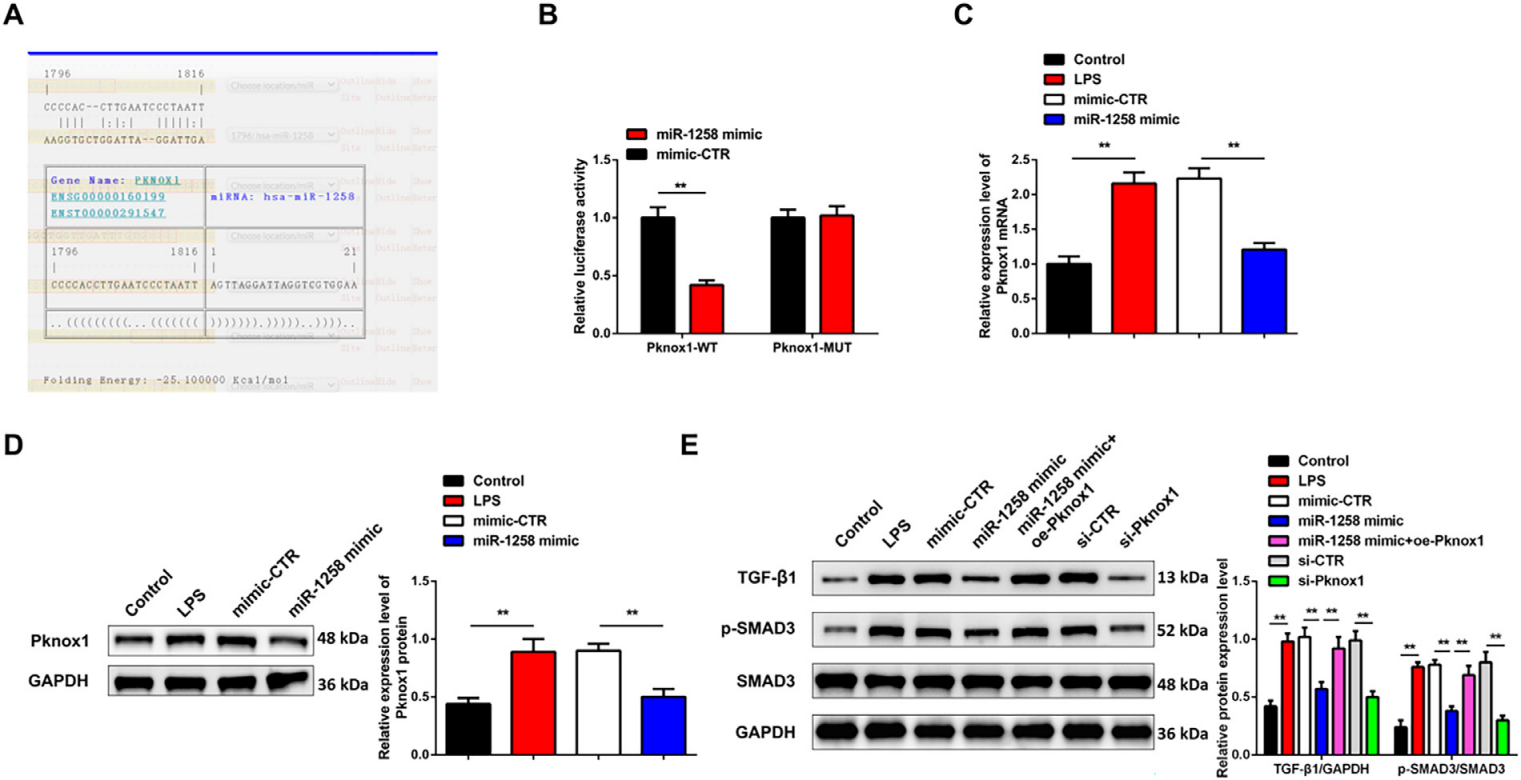
miR-1258 targets Pknox1 to control TGF-β1/SMAD3 cascade. The binding site of miR-1258 and Pknox1 (A), targeting the relationship between miR-1258 and Pknox1 (B), Pknox1 mRNA, and protein expression (C‒D), TGF-β1/SMAD3 cascade-related factor protein expression (E); Repetitio *n =* 3; Data were presented as mean ± standard deviation.

Pknox1 contains a conserved binding site for miR-1258 in the https://cm.jefferson.edu/rna22/ (Fig. 3A). Subsequent investigation indicated that miR-103a-3p mimics significantly decreased PKnox1-WT luciferase activity, whereas did not impact PKnox1-MUT luciferase activity (Fig. 3B). RT-qPCR and Western blot results confirmed Pknox1 expressions elevated in LPS and mimic-CTR transfected cells compared to miR-1258 mimic and control cells (Fig. 3C,D). Further, when exploring the downstream mechanisms of the miR-1258 and Pknox1 in septic ALI, western blotting confirmed a significant increase of TGF-β1 and p-Smad3in LPS, mimic-CTR, miR-1258 mimic+oe-Pknox1, and si-CTR compared to the miR-1258 mimic and si-Pknox1 transfected cells (Fig. 3E). These results confirmed that miR-1258 targets Pknox1 to control TGF-β1/SMAD3 cascade.

### MiR-1258 prevents LPS-induced inflammation and oxidative stress in vivo

**Fig. 4:**
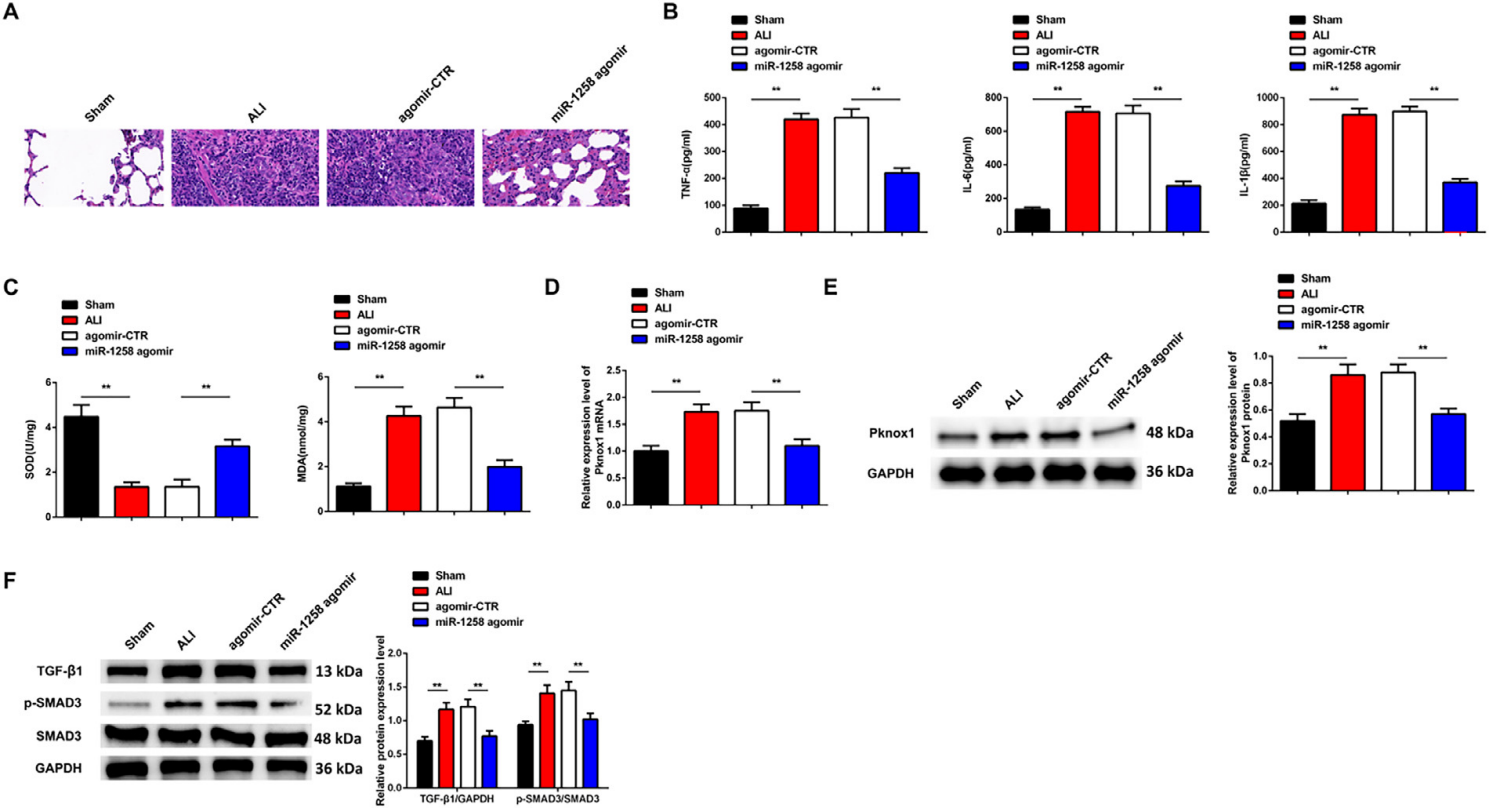
miR-1258 prevents LPS-induced inflammation and oxidative stress *in vivo*. H&E staining images of lung tissue (× 200, A), inflammatory factors in serum (B), oxidative stress-related indicators in lung tissue (C), Pknox1 mRNA and protein expression in lung tissue (D‒E), TGF-β1/SMAD3 cascade-related factor protein expression of mice (F); *n =* 10; Data were presented as mean ± standard deviation.

*In vivo,* studies were finally done to confirm the role of miR-1258 in LPS-induced inflammation. H&E tissue staining depicted no effect on the lung pathology in the sham group, as reflected by intact lung tissue structure and no alveolar septum edema and inflammation. However, LPS injection severely damaged alveolar structures and widened alveolar septa, accompanied by pulmonary interstitial exudation, hemorrhage, and massive inflammatory cell infiltration. However, these pathological damage observations in the ALI group were alleviated in miR-1258 agomir pre-treatment, as shown in Fig. 4A.

TNF-α, IL-6, and IL-1β expressions increased in ALI and agomir-CTR compared to the miR-1258 agomir and the sham groups shown in Fig. 4B. The SOD expression was significantly reduced in the ALI and Agomir-CTR-treated groups compared to the miR-1258 agomir-treated cells. However, the MDA expression was significantly increased in the ALI and Agomir-CTR but reduced in the miR-1258 agomir-treated groups (Fig. 4C). The RT-qPCR and western blot assays were used to analyze Pknox1 expression. The observations confirmed significantly increased Pknox1 mRNA and protein expression in ALI and agomir-CTR compared to miR-1258 agomir and sham group, as shown in Fig. 4D‒E. Western blot confirmed increased TGF-β1 and p-Smad3 protein expression in ALI and agomir-CTR but reduced TGF-β1 and p-Smad3 protein expressions in the miR-1258 agomir groups, as shown in Fig. 4F. These observations confirmed that miR-1258 prevents LPS-induced inflammation and oxidative stress *in vivo.*

## Discussion

The authors reported for the first time that overexpressing miR-1258 alleviated septic ALI by suppressing oxidative stress and inflammation by targeting Pknox1 to inactivate the TGF-β1/SMAD3 cascade, which provides a potential therapeutic strategy for the clinical treatment of severe septic infection. Sepsis is primarily caused by the cellular response to infection.^25^ The lung is an important immune organ and the first organ affected by sepsis.^26^ ALI caused by sepsis has the highest morbidity and mortality among the causes of ALI.^27^ The occurrence of sepsis is related to the bacteria or bacterial toxin LPS on the wound. LPS can induce sepsis, septic shock, and multiple organ dysfunction syndrome. LPS induces the body's immune response through a variety of signal transduction pathways and stimulates immune cells to produce a large number of inflammatory cytokines with thermogenic effects, such as TNF-a and IL-6, resulting in excessive activation of the immune system.^28^ Meanwhile, sepsis leads to the destruction of the alveolar epithelium, inflammatory exudation and respiratory distress. These phenomena are consistent with those observed in LPS-induced ALI mice in this study.^29^ In this study, the authors found that LPS injection induced severe destruction of alveolar structure, widening of alveolar interval, interstitial exudation, bleeding, and infiltration of inflammatory cells in mice. The levels of inflammatory and oxidative stress markers in the blood of the LPS-induced ALI mice increased significantly. This indicates that the in vivo model used in this study is highly representative and simulative.

Multiple miRNAs are dysregulated in ALI, covering various pathological and physiological processes, such as inflammation and oxidative stress.^30^^,^^31^ miR-144-3p enhances lung tissue damage, inflammation, and apoptosis in septic ALI mice.^32^ miR-34a knockdown attenuates oxidative stress and inflammation in septic ALI mice,^23^ and miR-217 regulates inflammation and oxidative stress and lung injury in septic mice.^33^ Therefore, studying the functions of aberrantly expressed miRNAs in septic ALI may contribute to developing effective treatments for ALI. In the present study, miR-1258 was downregulated in serum samples of septic ALI patients, LPS-treated mice, and the BEAS-2B cell model, suggesting that miR-1258 may be involved in the development of septic ALI. The present study's *in vitro* cell experiments showed that upregulating miR-1258 could alleviate LPS-induced cell damage by inhibiting oxidative stress and inflammatory insult, increasing cell viability, and inhibiting apoptosis. In addition, the results of animal experiments were consistent with the cellular observations.

Next, the authors explored the potential mechanism by which miR-1258 regulated septic ALI and focused on Pknox1. PKnox1 is a homeodomain transcription factor of the TALE superclass essential for embryogenesis.^34^ According to the previous investigations, PKnox1 cooperates with HOX/PBX complex in vitro and regulates Tp53 and Bcl-x, thus inhibiting oncogenic pathways via hindering MEIS1-dependent transcriptional co-activators recruitment.^35^ In addition, Pknox1 stimulates the expression of pro-inflammatory cytokines in aortic endothelial cell models^36^ and could regulate inflammatory diseases and organ damage, such as viral myocarditis^37^ and steatohepatitis.^38^ In the setting of septic ALI, it was confirmed that LPS treatment promoted Pknox1 expression, whereas up-regulation of miR-1258 did the opposite, further confirming Pknox1 as a downstream target of miR-1258. Furthermore, Pknox1 knockdown had similar effects to miR-1258 overexpression on LPS-treated cells, whereas Pknox1 overexpression could partially reverse the amelioration of LPS-treated cells damage by up-regulation of miR-1258.

Notably, the authors found the activated TGF-β1/SMAD3 cascade in LPS-treated BEAS-2B cells, while up-regulating miR-1258 or down-regulating Pknox1 suppressed TGF-β1 and p-Smad3 expressions. TGF-β1/SMAD3 cascade is involved in lung injury in inflammation, fibrosis, and epithelial-mesenchymal transition.^39^ Fei et al. have validated the alleviating effect of TGF-β1/SMAD3 cascade inactivation on septic ALI.^40^ Consistent with their findings, the authors confirmed that miR-1258 ameliorated LPS-induced oxidative stress and inflammation by targeting Pknox1 to inactivate the TGF-β1/SMAD3 cascade.

The study also has some limitations. The number of samples is insufficient, and larger populations need to be tested to elucidate the relationship between miR-1258 expression in serum and the pathological features of septic ALI. MiR-1258 may also exert its protective role in ALI by regulating other downstream targets, which requires further studies to detect and identify alternative miR-1258 targets involved in ALI progression.

## Conclusion

In evidence, miR-1258 ameliorates septic ALI by suppressing inflammation and oxidative stress by suppressing Pknox1 expression and TGF-β1/SMAD3 cascade activation. The findings of this study. This study demonstrates the clinical relevance of the functional relationship of miR-1258 in patients with sepsis and gives a novel basis for targeting Knox1 and its downstream molecular axis as a possible alternative for developing effective drug therapy against ALI. The present findings contribute to further understanding of septicaemia-induced organ damage and provide a new perspective for the diagnosis and treatment of septicaemia-induced lung injury based on miRNA. Further clinical trial data are required to validate the obtained preliminary in vitro and in vivo results.

## Authors' contributions

XiaoMeng Xu designed the research study. XiaoMeng Xu performed the research. XiaoHong Xu provided help and advice on the experiments. JinLiang Cao analyzed the data. XiaoMeng Xu, XiaoHong Xu and LuoYang Ruan wrote the manuscript. All authors contributed to editorial changes in the manuscript. All authors read and approved the final manuscript.

## Funding

Not applicable.

## Ethical Statement

The experiment research protocol was approved by the Ethics Committee of Guangzhou Hospital of Integrated Traditional and West Medicine and all experimental procedures conformed with institutional guidelines, and all patients participating in this study provided written informed consent in accordance with the “Helsinki Declaration”.

The use of all animals was approved by the experimental Ethics Committee of Guangzhou Hospital of Integrated Traditional and West Medicine (Approval NO. GZITWM201903IK503). All animal experiments complied with the ARRIVE guidelines and performed in accordance with the National Institutes of Health Guide for the Care and Use of Laboratory Animals.

## Availability of data and materials

The data and materials used to support the findings of this study are available from the corresponding author.

## Informed consent

Informed consent was obtained from all individual participants included in the study.

## Conflicts of interest

The authors declare no conflicts of interest.

## References

[1] Zhang J, Wang C, Wang H, Li X, Xu J, Yu K (2021). Loganin alleviates sepsis-induced acute lung injury by regulating macrophage polarization and inhibiting NLRP3 inflammasome activation. Int Immunopharmacol.

[2] Xu Q, Wang J. (2020). IGFBP7 aggravates sepsis-induced acute lung injury by activating the ERK1/2 pathway. Folia Histochem Cytobiol.

[3] Costa EL, Schettino IA, Schettino GP. (2006). The lung in sepsis: guilty or innocent?. Endocr Metab Immune Disord Drug Targets.

[4] Tomita K, Saito Y, Suzuki T, Imbaby S, Hattori K, Matsuda N (2020). Vascular endothelial growth factor contributes to lung vascular hyperpermeability in sepsis-associated acute lung injury. Naunyn Schmiedebergs Arch Pharmacol.

[5] Jiang WY, Ren J, Zhang XH, Lu Z-L, Feng H-J, Yao X-L (2020). CircC3P1 attenuated pro-inflammatory cytokine production and cell apoptosis in acute lung injury induced by sepsis through modulating miR-21. J Cell Mol Med.

[6] Bechara RI, Pelaez A, Palacio A, Joshi PC, Hart CM, Brown LAS (2005). Angiotensin II mediates glutathione depletion, transforming growth factor-beta1 expression, and epithelial barrier dysfunction in the alcoholic rat lung. Am J Physiol Lung Cell Mol Physiol.

[7] Wagener BM, Hu M, Zheng A, Zhao X, Che P, Brandon A (2016). Neuronal Wiskott-Aldrich syndrome protein regulates TGF-β1-mediated lung vascular permeability. FASEB J.

[8] Akbarshahi H, Sam A, Chen C, Rosendahl AH, Andersson R. (2014). Early activation of pulmonary TGF-β1/Smad2 signaling in mice with acute pancreatitis-associated acute lung injury. Mediators Inflamm.

[9] Xia W, Pan Z, Zhang H, Zhou Q, Liu Y. (2020). Inhibition of ERRα aggravates sepsis-induced acute lung injury in rats via provoking inflammation and oxidative stress. Oxid Med Cell Longev.

[10] Chen H-Y, Ho Y-J, Chou H-C, Liao E-C, Tsai Y-T, Wei Y-S (2020). TGF-β1 signaling protects retinal ganglion cells from oxidative stress via modulation of the HO-1/Nrf2 pathway. Chem Biol Interact.

[11] Hu F, Dong X, Li W, Lv J, Lin F, Song G (2021). miR‑351‑5p aggravates lipopolysaccharide‑induced acute lung injury via inhibiting AMPK. Mol Med Rep.

[12] Luo Q, Zhu J, Zhang Q, Xie J, Yi C, Li T. (2020). MicroRNA-486-5p promotes acute lung injury via inducing inflammation and apoptosis by targeting OTUD7B. Inflammation.

[13] Suo T, Chen GZ, Huang Y, Zhao K-C, Wang T, Hu K (2018). miRNA-1246 suppresses acute lung injury-induced inflammation and apoptosis via the NF-κB and Wnt/β-catenin signal pathways. Biomed Pharmacother.

[14] Lin J, Lin Z, Lin L. (2021). MiR-490 alleviates sepsis-induced acute lung injury by targeting MRP4 in new-born mice. Acta Biochim Pol.

[15] Ling Y, Li Z-Z, Zhang J-F, Zheng X-W, Lei Z-Q, Chen R-Y (2018). MicroRNA-494 inhibition alleviates acute lung injury through Nrf2 signaling pathway via NQO1 in sepsis-associated acute respiratory distress syndrome. Life Sci.

[16] Hwang J-S, Jeong E-J, Choi J, Lee Y-J, Jung E, Kim S-K (2019). MicroRNA-1258 inhibits the proliferation and migration of human colorectal cancer cells through suppressing CKS1B expression. Genes.

[17] van der Sijde F, Vietsch EE, Mustafa DAM, Li Y, van Eijck CHJ. (2020). Serum miR-338-3p and miR-199b-5p are associated with the absolute neutrophil count in patients with resectable pancreatic cancer. Clin Chim Acta.

[18] Braga EA, Loginov VI, Burdennyi AM, Filippova EA, Pronina IV, Kurevlev SV (2018). Five hypermethylated MicroRNA genes as potential markers of ovarian cancer. Bull Exp Biol Med.

[19] Milara J, Peiro T, Serrano A, Cortijo J. (2013). Epithelial to mesenchymal transition is increased in patients with COPD and induced by cigarette smoke. Thorax.

[20] Bernard GR, Artigas A, Brigham KL, Carlet J, Falke K, Hudson L (1994). The American-European Consensus Conference on ARDS. Definitions, mechanisms, relevant outcomes, and clinical trial coordination. Am J Respir Crit Care Med.

[21] Fang Y, Xiao C, Wang L, Wang Y, Zeng J, Liang Y (2023). Synergistic enhancement of isoforskolin and dexamethasone against sepsis and acute lung injury mouse models. J Inflamm Res.

[22] Tu G-W, Shi Y, Zheng Y-J, Ju M-J, He H-Y, Ma G-G (2017). Glucocorticoid attenuates acute lung injury through induction of type 2 macrophage. J Transl Med.

[23] Chen S, Ding R, Hu Z, Yin X, Xiao F, Zhang W (2020). MicroRNA-34a inhibition alleviates lung injury in Cecal ligation and puncture induced septic mice. Front Immunol.

[24] Yao W, Xu L, Jia X, Li S, Wei L. (2021). MicroRNA‑129 plays a protective role in sepsis‑induced acute lung injury through the suppression of pulmonary inflammation via the modulation of the TAK1/NF‑κB pathway. Int J Mol Med.

[25] Zhang Y, Yu W, Han D, Meng J, Wang H, Cao G. (2019). L-lysine ameliorates sepsis-induced acute lung injury in a lipopolysaccharide-induced mouse model. Biomed Pharmacother.

[26] Kumar V. (2020). Pulmonary innate immune response determines the outcome of inflammation during pneumonia and sepsis-associated acute lung injury. Front Immunol.

[27] Chen J, Li C, Liang Z, Li C, Li Y, Zhao Z (2021). Human mesenchymal stromal cells small extracellular vesicles attenuate sepsis-induced acute lung injury in a mouse model: the role of oxidative stress and the mitogen-activated protein kinase/nuclear factor kappa B pathway. Cytotherapy.

[28] Wang B, Sun Q, Ye W, Li L, Jin P (2021). Long non-coding RNA CDKN2B-AS1 enhances LPS-induced apoptotic and inflammatory damages in human lung epithelial cells via regulating the miR-140-5p/TGFBR2/Smad3 signal network. BMC Pulm Med.

[29] Maybauer MO, Maybauer DM, Fraser JF, Szabo C, Westphal M, Kiss L (2010). Recombinant human activated protein C attenuates cardiovascular and microcirculatory dysfunction in acute lung injury and septic shock. Crit Care.

[30] Liu H, Chen X, Gao W, Jiang G. (2012). The expression of heparanase and microRNA-1258 in human non-small cell lung cancer. Tumour Biol.

[31] Lu Z, Feng H, Shen X, He R, Meng H, Lin W (2020). MiR-122-5p protects against acute lung injury via regulation of DUSP4/ERK signaling in pulmonary microvascular endothelial cells. Life Sci.

[32] Xu R, Shao Z, Cao Q. (2021). MicroRNA-144-3p enhances LPS induced septic acute lung injury in mice through downregulating Caveolin-2. Immunol Lett.

[33] Yan J, Yang F, Wang D, Lu Y, Liu L, Wang Z. (2021). MicroRNA-217 modulates inflammation, oxidative stress, and lung injury in septic mice via SIRT1. Free Radic Res.

[34] Purushothaman D, Blasi F. (2018). The genetics and the molecular functions of the PREP1 homeodomain transcription factor. Int J Dev Biol.

[35] Bisaillon R, Wilhelm BT, Krosl J, Sauvageau G. (2011). C-terminal domain of MEIS1 converts PKNOX1 (PREP1) into a HOXA9-collaborating oncoprotein. Blood.

[36] Cimmino I, Prisco F, Orso S, Agognon AL, Liguoro P, De Bias D (2021). Interleukin 6 reduces vascular smooth muscle cell apoptosis via Prep1 and is associated with aging. FASEB J.

[37] Gou W, Zhang Z, Yang C, Li Y. (2018). MiR-223/Pknox1 axis protects mice from CVB3-induced viral myocarditis by modulating macrophage polarization. Exp Cell Res.

[38] Oriente F, Cabaro S, Liotti A, Longo M, Parrillo L, Pagano TB (2013). PREP1 deficiency downregulates hepatic lipogenesis and attenuates steatohepatitis in mice. Diabetologia.

[39] Ma K, Li C, Xu J, Ren F, Xu X, Liu C (2020). LncRNA Gm16410 regulates PM(2.5)-induced lung endothelial-mesenchymal transition via the TGF-β1/Smad3/p-Smad3 pathway. Ecotoxicol Environ Saf.

[40] Xu F, Lin S-H, Yang Y-Z, Guo R, Cao J, Liu Q. (2013). The effect of curcumin on sepsis-induced acute lung injury in a rat model through the inhibition of the TGF-β1/SMAD3 pathway. Int Immunopharmacol.

